# Mild cognitive impairment: The dilemma

**Published:** 2009-01

**Authors:** Charles Pinto, Alka A. Subramanyam

**Affiliations:** Department of Psychiatry, TN Medical College and BYL Nair Hospital, Mumbai - 400008, India

**Keywords:** Cognition, criteria, diagnosis, medications, memory, nomenclature, risk factors

## Abstract

Memory complaints are ubiquitous in our aging population. Many older adults fear that today’s forgetfulness will usher in tomorrow’s dementia. Mild cognitive impairment (MCI) is considered by many as an intermediary stage for dementia. Though the nomenclature has been varied and extensive, the criteria by the American Academy of Neurology and the EADC have been helpful. Prevalence rates varying from 3% to as high as 59% with a conversion rate to dementia varying from 8 to 15% only increases the need for diagnostic tests and markers which are in the form of neuropsychological tests, neuroimaging and other biological markers.

Medications indicated for treatment of mild to severe Alzheimer’s Disease (AD) are offered to persons with MCI with a varying type of response which does not hold in the long run to newer strategies of exploring disease modifying drugs which hold a better promise. This benefit with management of risk factors like hypertension and diabetes coupled with non-pharmacological approaches like exercise and social networking has thrust upon us the necessity for coordinating our efforts to improve detection and management of MCI.

## INTRODUCTION

Mild cognitive impairment (MCI), most simplistically defined refers to cognitive changes in the absence of dementia. It has been likened to an intermediate stage between normalcy and dementia. Indeed the entity probably stemmed from the pursuit of clinicians to try and find the missing piece of the puzzle between so called “normal” elderly and the elder with dementia.

Reisberg in 1988[1] first described an entity with Global Deterioration Scale Score (GDS) of 3. Subsequently, Flicker and colleagues[2] wrote an article on patients at risk for dementia, also with GDS scores of 3. Of course, it was Peterson in 1997,[3] who then termed this entity as Mild Cognitive Impairment or MCI, wherein in the absence of a rating scale clinical suspicion would arise when certain criteria were met (elaborated later).

While many researchers will argue that it is indeed MCI which should be labeled as the prodrome to Alzheimer’s Disease (AD), evidence shows that not all MCI progresses to Alzheimer’s Dementia. Of those that do, the benefits of early treatment were transient.

This brings up a host of controversial questions: Is MCI a scientist’s nomenclature alone or a true biological entity? Will the knowledge of it then change our approach to treatment? Will it be ethical to treat the group supposed to be at risk? Are we merely splitting hairs or will investment into this field of research yield tangible benefits to the patients?

We hope this article will answer some of these questions.

## NOMENCLATURE

MCI has often been referred to by various names, the commonest of which have been including:

- Benign senescent forgetfulness (BSF)
- Age associated memory impairment (AAMI)
- Age related memory decline (ARMD)
- Age related cognitive decline (ARCD)
- Mild cognitive disorder/mild cognitive dysfunction (MCD)
- Mild cognitive impairment (MCI)
- Mild neurocognitive disorder (MND)
- Cognitive impairment no dementia (CIND)
- Questionable dementia (QD)
- Late life forgetfulness (LLF)

Of these the following have stood the test of time, and hence are defined:

- **BSF** - Individuals have poor retrieval of relatively minor details of an episode but no memory loss of the episode itself.
- **AAMI** - by NIMH is memory loss of 1 standard deviation on testing with no cognitive impairment in advancing age and normal intelligence.
- **ARCD** - is memory impairment as in AAMI and objective cognitive decline within normal limits for age now known as CIND (Cognitive Impairment Not Dementia)
- **MCI** - have definite memory and cognitive impairment with GDS score of 3 and a CDR score of 0.5 also known as questionable dementia.

These have at times been referred to as sub-types of MCI.[4] Recent efforts have been directed at developing a uniform diagnostic classification for MCI.

## PRESENTING SYMPTOMATOLOGY

It was Peterson who first described the concept of MCI in 1997,[5] an entity which has grown since then. He described it along a continuum between normal ageing and dementia, and the criteria as defined by him, which were subsequently modified. Both the criteria are:

**Table:** 

1997- Original	1999- Modified
Memory complaint Objective memory disorder Absence of other cognitive disorders or repercussions on daily life Normal general cognitive function Absence of dementia	Memory complaint Objective memory disorder Absence of other cognitive disorders or repercussions on daily life Normal general cognitive function Absence of dementia
*6) 0.5 stage of clinical dementia rating scale (CDR)	

These criteria have been subject of wide debate time and again. The validity and reliability of the criteria have often been questioned. This ongoing dilemma and clinical usefulness of the criteria, led to the following description of MCI by the EADC[6] working group:

1. Cognitive complaint emanating from the patient and/or his/her family,
2. The subject and/or informant report a decline in cognitive functioning relative to previous abilities during the past year,
3. Cognitive disorders evidenced by clinical evaluation: impairment in memory and/or another cognitive domain,
4. Cognitive impairment does not have major repercussions on daily life. However, the subject may report difficulties concerning complex day-to-day activities,
5. No dementia.

Thereby this is more of a clinical diagnosis, with less emphasis on memory. The EADC working group thus brought forth the concept of the “*MCI Syndrome*”- thereby combining the clinical picture supported by neuropsychological testing, and not either one in isolation.

The American Academy of Neurology,[7] along the same lines, but a tad more simplistically, has defined MCI as:

1. Memory complaints preferably corroborated by an informant
2. Objective memory impairment
3. Normal general cognitive functioning
4. Intact activities of daily living (ADL)
5. Not demented

A study done in Mumbai by Gambhire and Pinto[8] revealed that almost all patients who had MCI (as compared to normal’s and patients with dementia reported forgetfulness). The scores on the NPI-inventory, as well as NPI-distress scale were in between normals and patients with dementia, and this was of statistical significance. Further, analysis of the symptoms revealed that patients with MCI reported more depression, anxiety and apathy, as compared to those with dementia, who reported more of delusions, agitation, irritability and altered night time behavior.[8] We probably need larger studies focusing on the behavioral symptoms in MCI, and their predictability in progression to dementia.

## SUBTYPES OF MCI[9]

### A) Based on cognitive features

This can be further divided into:

1. Amnestic MCI - Predominant impairment in memory
2. Multiple domain MCI: Impairment noticed in more than one cognitive domains which can include memory
3. Single-domain non-amnestic MCI: Predominant impairment in any one cognitive domain apart from memory

1 and 2 have an equal risk of progress to AD.

### B) Based on aetiopathology

These can be of the following types:

1. Neurodegenerative: (MCI, Pre-Alzheimer’s, Lewy Body, Fronto-temporal or focal atrophy)
2. Vascular: Vascular dementia and mixed dementia
3. Dysthymia or dysphoria (anxious and/or depressed states)

## EPIDEMIOLOGY

The rates of MCI reported vary from 3 to 17%[10] it being 3% at 60 years to 15% at the age of 75.[11] In fact, the rate of development of MCI was about 5.3% per year[11] (3.5% in the seventh decade of life and 7.2% in the eighth decade). Men seemed to be more affected than women (though we must remember at this point that AD is more seen in women).[11]

Among the early Indian studies done for dementia, the one done in 2001 by Vas, Pinto *et al*. showed a prevalence of 0.25% Alzheimer’s disease in the population with it increasing to 1.5% for those 65 years and older.[12] However, MCI was not studied then.

Recently, an Indian study from Calcutta[13] showed prevalence of 14.89% MCI-of which the amnestic type (more seen in men) was 6.04% and the multiple domain type (more seen in men) was 8.85%. The study from Cochin[14] also reported the prevalence to be about 14.89%.

Satishchandra and group[15] on the other hand reported an incidence in a clinical setting to be as high as 47.1%. Mridula, Alladi *et al*.[16] in their clinic sample reported a rate of 59% with MCI, in their elder population.

Conversion rates from MCI to AD were found to be 10-15%[11] as reported by Peterson in his sample at a specialty clinic, whilst it was 8-10%[11] in the general population, by his estimate. Mridula, Alladi *et al*. in their clinic sample, showed 11% conversion rate[16] to AD, during a 13 month follow up.

## ASSESSMENT

By definition and the clinical presentation, one can easily conclude that memory testing would be the most important single means of accurately assessing MCI. However, it becomes essential to have a comprehensive set of tests to accurately reach the suspicion that an individual may have MCI. Of historical importance are the CAMDEX[17] (Cambridge Mental Disorders of the Elderly Examination) where “minimal dementia” is suggestive of MCI, and “limited cognitive disturbance” on the CARE[18] (Comprehensive Assessment and Referral Evaluation).

Although presently there is no clear guideline, the following are the commonly administered tests to aid the diagnosis:

### 

#### Mini mental status examination (MMSE)[19]

It is probably the most widely used test for bedside memory testing. Interpretation, however, can pose to be an issue. It has both high floor and ceiling effect. A clinician should be alerted to scores of 26 (in the non-educated) and 28 (in the educated) for further assessment, follow up and surveillance for MCI.

### Clinical dementia rating scale (CDR)[20]

The score of 0.5 on this scale is of diagnostic importance for MCI, according to Peterson’s modified criteria. Largely debated, including by Ganguli and co-workers, the American Academy of Neurology (AAN) does accept this score as equivalent to the presence of MCI. Occasionally, people with MCI can acquire a score of 0 on MCI.

### Intelligence testing

The individual undergoing testing should have average intellectual functioning.

### Global deterioration scale (GDS)[21]

A score of 3 is considered indicative of MCI.

#### Neuropsychiatric inventory (NPI) and short form (NPI-Q)[22]

Presence of psychiatric symptoms and caregiver distress can be assessed.

### Informant questionnaire on cognitive decline in the elderly (IQ-CODE)[23]

It addresses the objective reporting by the caretaker on the day-to-day behavior of the person. It requires a very well informed caregiver for the same.

To test progression of the problem, if any, the following tests are also used in addition to the above:

### Clock drawing test[24]

In educated persons, this very simple test can test progression of basic cognition and abilities. It is a bedside test, used frequently by many researchers.

### Tests for verbal fluency and verbal memory

Verbal fluency and word list memory tasks (both immediate and delayed) are useful and simple tools to monitor and follow up suspected cases. These tests are also used during PET activation procedures.

It is of course, not possible to use all tests, however, as far as possible, these tests give a good guideline for assessment of MCI.

## PATHOLOGY

**Gross pathological changes:** It is only in patients where progression from MCI to AD occurs, that the gross pathology[25] can be accurately assessed. In these patients, there is evidence of neuritic plaques and neurofibrillary tangles (particularly in the entorrhinal cortex and hippocampus), along with amyloid deposition. Braak and Braak in autopsies conducted on a number of patients in various stages of cognitive change demonstrated that plaques and tangles are seen prior to the appearance of amyloid, and this might be significant while following up patients of MCI to AD.

**Microscopic changes:**[26,27] There are a variety of microscopic changes which are:

1. Neuronal loss:[26] Decreased neuron number and volumes are seen mainly in the entorrhinal cortex (which forms a nerve relay) and hippocampus (memory storage).
2. Beta-amyloid deposition: Though the deposition of beta-amyloid in MCI is intermediate between normal elderly and those with AD, this is not of statistical significance.
3. Neurofibrillary tangles and tau proteins:[27] Phosphorylated tau protein within the neurofibrillary tangle is seen in the vulnerable regions.[28,29] The presence of amyloid does tend to accelerate the tangle formation.
4. Down regulation of trkA RNA: There is evidence that both those with MCI and those with AD had significant loss in the number of trkA-containing neurons,[30] (46% decrease for MCI, and 56% for AD). The alterations in the number of nucleus basalis neurons containing trkA immunoreactivity occurs early and are not accelerated from the transition from MCI to mild AD.
5. Loss of Choline Acetyl Transferase (ChAT):[31] In individuals with MCI and mild AD, ChAT activity was unchanged in the inferior parietal, superior temporal and anterior cingulate cortices. In contrast, ChAT activity in the superior frontal cortex was significantly elevated above normal controls in MCI subjects, whereas the mild AD group was not different. Hippocampal ChAT activity was significantly higher in MCI subjects. The up regulation in frontal cortex and hippocampal ChAT activity could be an important factor in preventing the transition of MCI subjects to AD.

## BIOLOGICAL MARKERS

The advent of a prodrome to Alzheimer’s disease, and the possibility of early intervention, led to a number of researchers looking for tangible, quantifiable means of assessing the changes. The most commonly studied biomarkers can be divided into the following groups:

1. Related to the gross pathological changes of neurofibrillary tangles and amyloid sheets, are the cerebrospinal fluid biomarkers (the 42 amino acid form of beta-amyloid -A beta, total tau, and phospho tau). Recent advances further support a notion that plasma A-beta levels, expressed as an A beta-42/A beta 40 ratio[32] could also be of value particularly in the progression of MCI to AD.
2. Another biomarker of importance, related to lipid peroxidation is Isoprostane[33] which is found elevated in the urine, CSF and blood of AD patients. It has promising value in conversion of MCI to AD.
  In a 2-year longitudinal study of MCI patients and normal controls, Deleon *et al*. showed that relative to controls, MCI patients showed decreased memory and hippocampal volumes and elevated CSF levels of hyperphosphorylated tau and isoprostane.[33]
3. Measurement of a protein bio-marker complex[34] that may include one of a transthyretin protein and/or a prostaglandin-H2 D-isomerase protein, and at least one second, different protein.
4. In AD and MCI patients, there is a significant rise in the percentage of monocytes producing cytokines[35] (IL-1β, IL-6, IL-12 and TNF-α), as well as a decreased response of these cells to inflammatory challenges, when compared with controls.

## GENETIC MARKERS

The complex genetics which AD and also MCI are proving to have makes it difficult to pinpoint a single gene or allele which may be responsible. Many researchers have put forth their various hypotheses.

1. In cognitively impaired patients without dementia, the utility of Apo lipoprotein E (ApoE) genotyping is unclear. What is known is that ApoE epsilon 4 carrier status was associated with conversion to AD. Its role in MCI may be limited.[36]
2. Lindsay Farrer and colleagues have data collected from the Multi-Institutional Research in Alzheimer’s Genetic Epidemiology (MIRAGE) study, which supports the idea that risk factors for vascular disease and AD are common. One of the families of genes they have investigated is those that encode paraoxonase (PON - an enzyme that is expressed in the liver) which show polymorphisms in MCI.[37]
3. The same group of researchers[37] is studying the Wadi Ara tribe in Israel, who has a high incidence of AD, perhaps due to inbreeding. They are studying polymorphisms on the ACE gene, and they believe that though this is more predictive of vascular dementia - this cascade of the A-beta amyloid is accelerated through the vascular events.[37]
4. Matay and colleagues from the National Institute of Mental Health (NIMH) have a unique technique of research called as “Imaging Genetics”.[38] They use imaging techniques to capture genetic polymorphisms. Their main area of work revolves around catechol-O-methyltransferase (COMT) and brain-derived neurotrophic factor (BDNF) on age-related changes in cognition. The met allele of a frequent polymorphism (val66met) in the gene for BDNF if present shows reduced hippocampal use during memory processing.
5. Giulio Pasinetti and his team use cDNA microarray and proteomic technologies[39] to assess and quantify changes in cognition.

## NEUROIMAGING

### A) Structural

1. MRI: Reductions in the medial temporal cortex particularly the entorrhinal and hippocampal volumes are well-established risk factors in AD. However, in MCI, their predictive value is yet to be evidenced.[40]

### B) Functional

1. Magnetic resonance spectroscopy (MRS): Effect of cognitive changes on the metabolite ratios is the principle of assessment of the MRS. A host of researchers have found reduction in the N-acetylaspartate to creatinine ratio particularly in the temporal and parietal lobes in AD. Also an elevation of myoinositol is seen. However, the evidence in MCI is yet lacking.[41] Some researchers have been able to show posterior cingulate involvement[42] in MCI.
2. Diffusion tensor imaging (DTI): This method produces in vivo images of biological tissues weighted with the local microstructural characteristics of water diffusion. Higher diffusivity in the left centrum semi-ovale, left and right temporal regions and left hippocampal region[43] is seen in cognitively affected subjects. The combination of MRI and DTI hold promise in the future.[44]
3. Diffusion weighted imaging (DWI): Diffusion imaging makes use of the variability of “Brownian motion” of water molecules in brain tissue. Cell membranes, vascular structures, and axon cylinders, for example, limit or restrict the amount of diffusion. Also, chemical interactions of water and macromolecules affect diffusion properties. Therefore, in the brain, water diffusion is referred to as “apparent diffusion.” Higher Apparent Diffusion Coefficient’s (ADC’s) have been found in hippocampus, temporal lobe gray matter, amygdale, posterior cingulated and corpus callosum of patients with MCI compared with that of controls.[45]
4. Functional MRI (fMRI): Increased activation in the medial temporal lobe with decreasing activation in the postero-medial cortices is seen in fMRI.[46] The reverse was seen while performing fMRI after exposure to galantamine,[47] suggesting clear involvement of the cholinergic system in declining cognition. With 4T fMRI subjects with MCI showed decreased magnitude of activation in bilateral frontal cortex regions (during encoding and retrieval), the left hippocampus (during retrieval), and the left cerebellum (during encoding) compared with magnitude of activation in control subjects. Patients with mild cognitive impairment showed increased activation in the posterior frontal lobes (during retrieval). Lower hippocampal activation during retrieval was the most significant correlate of clinical severity of memory loss in mild cognitive impairment.
5. Positron emission tomography (PET) and single photon emission computerized tomography (SPECT): Reduced glucose metabolism (in PET) and reduced blood flow (in SPECT) are reported in the temporo-parietal association cortices, posterior cingulate and hippocampus in MCI. Similarly, gray matter loss in the entorhinal and hippocampal areas and hypometabolism or hypoperfusion in the posterior cingulate cortex and precuneus has also been observed.[48] The above have a predictive value in conversion of MCI to AD.[49]
  69% of Mridula, Alladi *et al*. sample showed abnormal brain imaging and vascular lesions in subjects with MCI.[16]

## TREATMENT ISSUES

### A) Pharmacological

Till date, no medication has been approved for use in MCI. However, the conversion of amnestic MCI to AD, has led to a surge of research in the field.

1. Acetylcholinesterase inhibitors (AChEI’s): There were two historical trials with AChEI’s- one using donepezil short term, and the other a long term study using galantamine and donepezil. The results indicated that AChEI’s have a transient effect on the conversion of MCI to AD, which does not hold up beyond 18 months.[50] Thus, the recommendation is that patients with amnestic MCI should be screened for the ApoE4 allele, and only if present should be given AChEI’s.[50] This suggests that the AChEIs have a symptomatic and potentially clinically significant effect, but one that is transient. Placebo-controlled trials have failed to show an effect of rivastigmine on conversion from MCI to dementia while galantamine was associated with some improvement on secondary measures of cognition but not with any effect on the conversion rate to dementia at 24 months. An unexpectedly high galantamine-associated death rate (or perhaps an unexpectedly low placebo-associated death rate) resulted in concern about the balance of galantamine’s risks and benefits for patients with MCI.
2. Memantine: Memantine has not been reported to benefit patients with MCI.
3. COX-2 inhibitors: Like rofecoxib have met with little benefit.[50]
4. Anti-amyloid therapies: Secretase inhibitors help reduce amyloid production by inhibiting the secretase activity. Similarly, fibrillogenesis inhibitors (alzhmed and cliniquinol) are being explored. Lastly, vaccines which would prevent amyloid plaque formation and actually aid regression are under scrutiny.[51]
5. Neurotonics: Like piracetam are highly debatable and have not met with any evidence.[50]
6. Oestrogens: The initial interest in post-menopausal women and also men has died down as it actually may increase the risk for MCI and AD.[52,53]
7. Antioxidants like Vitamin E, Vitamin C, Gingko biloba and curcumin (from turmeric) are hypothesized to reduce oxidative stress and ageing, yet work in this field is largely in the incipient stages.[54,55]
8. Dopamine agonists in the NIMHANS study using Piribedil a dopamine agonist. There was an improvement in global cognitive function in mild cognitive impairment significantly as compared to placebo after treatment with Piribedil.[56] Perhaps other dopaminergic agonists should be considered for appropriately designed research in patients with MCI.
9. Miscellaneous: Like CDP choline, omega 3 fatty acid, Ca channel blocker nimodipine and testosterone supplementation have shown to be partially effective.
10. Drugs on trial for MCI current testing for MCI include vasoactive intestinal peptide (AL-208), a selective metabotropic glutamate receptor antagonist (C-105), a novel L-type calcium channel blocker (MEM-1003), a phosphodiesterase inhibitor (MEM-1414), a g-aminobutyric acid B receptor antagonist (SGS-742), and a selective serotonin receptor (5HT6) antagonist (SGS-518).

### B) Non-pharmacological[57]

This will perhaps assume more importance in the near future.

1. Treatment of associated co-morbidities like sleep, depression, etc.
2. Treatment of vascular risk factors: Like hypertension, weight gain, hyperlipidemia, etc.
3. Social networking: Isolation exacerbates cognitive decline. Patients with MCI should be encouraged to socialize.
4. Cognitive activities and training: Cognitive activities like crossword puzzles, novels, su-doku, etc. all help against cognitive decline. Cognitive training as a specialized therapeutic intervention helps too.
5. Physical exercise: Although there is no clear evidence of the same in MCI specifically, physical exercise does improve cognitive ability, or definitely slows down decline.

### Other relevant issues

1. Informant reliability: The informant reliability is a matter of grave consideration in MCI. A recent study by Ozioma *et al*.[58] demonstrated a discrepancy on the financial domains of checkbook management, bank statement management, and bill payment, and on overall financial capacity, as reported by patients and informants, informants being more lax about the abilities. This could have important implications for case management and treatment.
2. Ethics: The diagnostic entity of MCI is one by exclusion. The validity and reliability of the entity still has to stand the test of time. Further, treatment becomes an ethical dilemma in view of the weak current evidence, and the apparent stigma attached to the same.[56]

## THE DILEMMA OF MCI: PRODROME OR DISEASE

MCI is still a controversial clinical concept, with several different diagnostic criteria and definitions of memory loss being used. Depending on the diagnostic criteria used, the prevalence and incidence of mild cognitive impairment vary considerably. It is thus best viewed as a somewhat heterogeneous clinical syndrome that for some patients may be a prodrome for dementia.

The challenge lies in the assessment of “significant impairment in functional ability,” which is one of the criteria for dementia. The most sensitive questions for detecting functional impairment in MCI are different from AD, and can be easily missed; for e.g., maintenance of their hobbies, ability to handle complex financial affairs, ability to use new equipment and tools, repetition of questions or forgetting the month and year.[55]

There is also controversy about the best way to objectively measure memory loss, whether by means of brief cognitive testing or a full neuropsychological evaluation. Another uncertainty is whether symptoms similar to BPSD are a useful prognostic sign in patients with MCI. Early recognition of the condition may prove to be the optimum point at which to intervene with preventive therapies when they become available.[57]

Unexpectedly high rates of MCI, widely considered to be a prodromal phase of Alzheimer’s disease (AD) and other dementias, have been found in a large population-based study of elderly patients. Presented recently at ICAD 2008: Alzheimer’s Association International Conference on Alzheimer’s Disease, the latest results from the Mayo Clinic Study of Aging show that study subjects developed MCI at a rate of about 5.3% per year and increased with advancing age — 3.5% per year for individuals aged 70 to 79 years and 7.2% per year for those 80 to 89 years said principal investigator Ronald C. Peterson “I think the implications of this constitute a major public health issue. As the baby boomers move into old age, we’re going to be talking about a lot of people who will develop mild cognitive impairment, which is presumably prodromal AD. It emphasizes the fact that we need to find disease-modifying therapies now, because we can’t afford to wait until this segment of the population ages into that period of significant risk.[59]

However, the growing use of the term as a diagnostic entity has empowered physicians and patients and has given them a more accurate view of the nature of the patient’s mild memory loss than was previously possible. A consensus regarding treatment issues and progress is yet to be arrived at. There is a need for longitudinal studies to validate any diagnostic approach. We lack studies, for instance, that demonstrate whether structural imaging will have an impact on the ultimate outcome of patients with MCI. Larger trials are needed to determine the efficacy of all treatments, both pharmacologic and non-pharmacologic. Indeed, there is a lot of scope for further development and progress in this field of research, both clinical and biological.
